# Endovascular Parent Artery Reconstruction for Ruptured Intracranial Blister Aneurysms: Demonstrating Safety and Efficacy After Early Flow Diversion

**DOI:** 10.7759/cureus.99536

**Published:** 2025-12-18

**Authors:** Nitin N Dange, Mayur Gharat, Nitin V Naikwade, Kushal Bhatia

**Affiliations:** 1 Neurosurgery, Seth Gordhandas Sunderdas Medical College and King Edward Memorial Hospital, Mumbai, IND; 2 Neurosurgery, Gleneagles Hospital, Mumbai, IND; 3 Neurosurgery, Lilavati Hospital and Research Centre, Mumbai, IND

**Keywords:** dual-antiplatelet therapy (dapt), flow diversion, parent artery reconstruction, ruptured blister aneurysms, subarachnoid hemorrhage

## Abstract

Background: Ruptured blister aneurysms are rare lesions that exhibit more aggressive behavior as compared to saccular aneurysms. Among all the endovascular treatment options, flow diversion seems to be showing the most promising results. Controversies regarding the optimal timing of flow diversion (early or delayed), along with the use and safety of dual antiplatelets, remain and are yet to be standardized.

Objective: The objective of this study was to assess the safety and efficacy of early endovascular flow diversion (within 72 hours) in ruptured intracranial blister aneurysms, specifically looking at the bleeding risk and treatment benefit of a standard DAPT regimen.

Methods: This study involved a retrospective analysis of patients with ruptured intracranial blister aneurysms at a tertiary care hospital in Mumbai, India (February 2017 - December 2022). All patients treated with endovascular embolization using a flow diverter device (FDD) within the first three days after rupture were included. A standard dual antiplatelet therapy (DAPT) protocol was used to prevent thromboembolic complications. Outcomes measured included complete angiographic obliteration and clinical outcome, mRS ≤2.

Results: Mean age was 58 (14 ± 8.36) years, and 57% (12/21) were women. The follow-up ranged from 12 to 30 months (mean 16.6 months). Out of 21 patients, 18 (85.7%) had immediate intraoperative stasis in the aneurysmal sac, with an immediate occlusion rate of 42.8% and complete obliteration in 19 (90.5%) patients at six months follow-up. Two patients (9.5%) developed intraluminal thrombus, which was managed successfully with rescue tirofiban, leading to a 0% rate of procedural mortality or further embolic issues. The DAPT protocol did not lead to any cases of re-rupture or bleeding complications. All patients reached sustained functional independence, gauged by a modified Rankin Scale score of 0 to 2, at the final follow-up, which had a mean duration of 16.6 months.

Conclusion: The concept of endoluminal parent artery reconstruction using a flow diverter device is promising for the treatment of ruptured intracranial blister aneurysms. Early flow diversion combined with strong clinical data from this series shows that a DAPT regimen is safe and necessary in this emergency situation, successfully reducing thromboembolic problems without immediately increasing the risk of hemorrhagic sequelae.

## Introduction

Blister aneurysms are rare lesions characterized by a thin, fragile wall and an absent/negligible neck. They make up 0.9-6.5% of internal carotid artery lesions and fewer than 2% of all intracranial aneurysms [1]. A hemispheric and broad appearance, arising from a non-branching area of an artery, erratic morphological changes on immediate angiographic follow-up, fragility, and a propensity to rupture or regrow are some of the distinctive features that set these lesions apart from saccular aneurysms [1,2]. Several mechanisms have been proposed in the pathogenesis, including atherosclerosis, hemodynamic stress related to hypertension, and arterial dissection creating a pseudoaneurysm [3,4].

They were first reported by Sundt and Murphey over 40 years ago [5]. In up to 65% of cases, they originate from the anteromedial carotid wall [6]. The posterior inferior cerebellar artery, the basilar trunk, and the anterior communicating artery are further uncommon locations of occurrence [7-9]. Blister aneurysms are more common on the right side, have a small female preponderance, and frequently affect patients at a younger age than their saccular counterparts [10,11]. The majority of these aneurysms develop over the supraclinoid carotid artery on the anteromedial surface, which is curved so that blood flow directly hits the arterial wall [6]. Aneurysm development is mostly caused by increased hemodynamic stress acting on a sclerotic artery segment that is already diseased.

Sometimes, blister aneurysms are not easily detected in the initial angiography but may show enlargement in size and shape from a small hemispherical protrusion to a large aneurysm in a short time after rupture. This type of aneurysm has friable walls, making the risk of intraoperative and postoperative bleeding high [12].

Various available treatment options are available; one such is the reconstructive technique, which includes two types: (ii) surgical and (ii) endovascular, which include direct suturing, wrapping, clip-wrapping, wrap-clipping, and primary clipping and primary coiling, telescopic stenting (stent-in-stent technique), stent-assisted/balloon-assisted coiling, and flow diverters, respectively. Another is the deconstructive technique, including blockage of the parent artery via endovascular or surgical methods, either with or without bypass operations [13]. Among all the different available endovascular approaches (including stent-assisted coiling, glue embolization, and covered stents), flow diversion seems to be showing the most promising results [14-16].

However, literature on flow diversion in the acute ruptured setting remains limited, especially in the context of developing countries with potential delays in diagnosis and intervention. When planning intraluminal flow diversion, two crucial choices in clinical practice are the timing of treatment and the antiplatelet regimen. In this article, we present our experience of managing such ruptured intracranial blister aneurysms during the early course (<3 days) after presentation, using an endovascular flow diverter device (FDD) and a dual antiplatelet treatment (DAPT) protocol, integrating our experience with recent global data [14].

The primary objective of this study was to determine the clinical and angiographic outcomes of patients treated with early endovascular FDD embolization in ruptured intracranial blister aneurysms. The secondary objective was to determine the perioperative, intraoperative, and postoperative complications.

## Materials and methods

This was a retrospective study including cases managed between February 2017 and December 2022 by a single hybrid neurosurgeon at Gleneagles Hospital, Mumbai, India. Data was collected from electronic medical records, which are recorded when a patient is admitted and discharged.

Study population

Inclusion criteria were acute subarachnoid hemorrhage (SAH) confirmed by non-contrast computed tomography (CT), radiologically identified blister aneurysm on CT angiography (CTA), magnetic resonance angiography (MRA), or rotational digital subtraction angiography (DSA), and definitive treatment by a single FDD placement within three days of rupture. Exclusion criteria included saccular aneurysms, fusiform aneurysms, traumatic lesions, dissecting aneurysms, and non-ruptured blister aneurysms.

Clinical evaluation and imaging protocol

Upon admission, the patient's neurological condition was evaluated according to Hunt and Hess (HH) [17], World Federation of Neurosurgical Societies (WFNS) hemorrhage scale [18], and modified Fisher grading [19]. This is a standard reporting technique, and authorization is not required. Imaging studies were done in the form of CT, CTA, MRA, and DSA. Three-dimensional rotational angiography was done in all patients to detect and understand the morphology of the aneurysms to plan the treatment strategy (feasibility of the procedure, expected anatomical challenges, and hardware requirements). DSA confirmed morphology consistent with blister aneurysms: small, hemispherical bulges along non-branching vessel segments, typically <3 mm, poorly demarcated, and without a classical neck.

Antiplatelet and anticoagulation protocol

Systemic heparinization was used for all procedures (5,000 IU intra-arterial heparin bolus initially, then followed by 1,000 IU intravenous boluses every hour during the surgery). Heparinization was discontinued after endovascular treatment. Patients were given loading doses of dual antiplatelets (Tab. ticagrelor 180 mg and Tab. aspirin 75 mg) at the time of induction of anesthesia. Tirofiban rescue therapy was only used in cases of flow-limiting in-stent stenosis or angiographically verified thrombus development. In case of intraluminal thrombus formation after FDD deployment, the patient received an intra-arterial bolus of Tirofiban (as per weight) followed by continuous infusion for 18 hours.

Postoperatively, all patients were managed as per standard institute protocols for the management of ruptured intracranial aneurysms. Patients were continued on a dual antiplatelet regimen of Tab ticagrelor 90 mg twice daily and Tab aspirin 75 mg once daily for six months, after which they were shifted to a single antiplatelet (aspirin 75 mg) regimen for six months.

Technique

All patients were treated with endovascular intervention using an FDD under general anesthesia via the transfemoral approach. Various FDDs, like Pipeline™ Embolisation Device (PED) (Medtronic plc, Galway, Ireland) in four cases, the DERIVO® 2heal® Embolisation Device (Acandis GmbH, Pforzheim, Germany) in 14 cases, and the Silk Vista Flow Diverter (Balt, Montmorency, France) in three cases, were used. No adjunctive coiling was done in our cohort. Three-dimensional (3D) rotational angiography was used to determine working angles, parent artery diameters at proximal and distal landing zones, and aneurysm dimensions. In all cases, the tri-axial system was utilized through femoral access; a 0.027" or 0.021" microcatheter, a 5/6 Fr intermediate or distal access catheter, and a 6 Fr 80-cm-long sheath were utilized in accordance with compliance with the chosen FDD size.

The size of the FDD was selected to be about 0.5 mm larger (oversizing) than the diameter of the expected proximal landing point. Intraoperative stasis of flow in the aneurysmal sac was observed in the runs taken 15 minutes post deployment of the FDD. A noncontrast CT of the brain was done postprocedure to look for signs of re-rupture or thromboembolic complications. 

Follow-up

Follow-up DSA was done at six months and at 12 months (for patients without complete obliteration at six months) post procedure to look for aneurysm obliteration. Long-term neurologic outcome in the form of the modified Rankin Scale (mRS) score was recorded. A satisfactory result and clinical safety criteria were defined as mRS ≤2.

Outcomes

Outcome measures as primary outcomes include complete angiographic aneurysm obliteration at 6-12 months. Secondary outcomes include immediate procedural stasis and clinical outcome measured by the mRS at the last follow-up, and procedure-related complications such as puncture site hematoma, hydrocephalus, re-rupture.

## Results

Baseline characteristics

A total of 21 consecutive patients with intracranial ruptured blister aneurysms were treated with endovascular flow diverters within the first 72 hours after the onset of subarachnoid hemorrhage. The clinical follow-up ranged from 12 to 60 months (mean 35.52 months). The mean age was 58 (14±8.36) years, and 57% (12/21) were women. All our patients had a lower-grade SAH (HH I-III). Hypertension was present in 13 patients, with six on irregular therapy. Eleven patients had type II diabetes; dyslipidemia was also common. Nine cases (42.8%) demonstrated coexistent atherosclerotic vessel wall irregularity on initial DSA. Baseline clinical and imaging characteristics of these 21 cases treated with the FDD are shown in Table 1. All patients had a good presenting Glasgow Coma Scale (GCS). No patients required an external ventricular drain/shunt for hydrocephalus. Blister aneurysm as the cause of SAH was established in 16 cases after noninvasive imaging. The remaining five cases were diagnosed after 3D rotational angiography. Seventeen blister aneurysms were located at the supraclinoid internal carotid artery (ICA), three at the basilar trunk, and one at the anterior communicating artery.

**Table 1:** Baseline clinical and imaging characteristics FDD: flow diverter device; ICA: internal carotid artery

Case number	Location of Aneurysm	FDD Size (mm)	Immediate Stadis	Immediate Complete Obliteration	Complication	Status at 6-month Follow-up
1	Right Supraclinoid ICA	4.5x15	Present	No	-	Complete obliteration
2	Right Supraclinoid ICA	4.5x20	Present	Yes	-	Complete obliteration
3	Left Supraclinoid ICA	4.5x15	Present	Yes	Intraluminal thrombus formation post FDD deployment	Complete obliteration
4	Right Supraclinoid ICA	4.5x20	Present	No	-	Complete obliteration
5	Left Supraclinoid ICA	4.5x20	No	No	-	Partial obliteration
6	Mid basilar Artery	3.5x15	Present	Yes	-	Complete obliteration
7	Left Supraclinoid ICA	4.5x30	Present	Yes	-	Complete obliteration
8	Right Supraclinoid ICA	5.0x15	No	No	-	Complete obliteration
9	Left Supraclinoid ICA	4.5x20	Present	Yes	-	Complete obliteration
10	Proximal basilar Artery	3.5x15	Present	No	-	Complete obliteration
11	Left Supraclinoid ICA	4.5x20	Present	No	-	Complete obliteration
12	Mid basilar Artery	3.0x15	Present	Yes	-	Complete obliteration
13	Right Supraclinoid ICA	4.5x20	Present	Yes	-	Complete obliteration
14	Right Supraclinoid ICA	5.0x15	Present	No	-	Complete obliteration
15	Left Supraclinoid ICA	4.5x15	Present	No	-	Complete obliteration
16	Left Supraclinoid ICA	4.5x15	Present	Yes	Intraluminal thrombus formation post FDD deployment	Complete obliteration
17	AComm Artery	3.0x20	Present	No	-	Complete obliteration
18	Left Supraclinoid ICA	3.5x15	No	No	-	Partial obliteration
19	Right Supraclinoid ICA	4.5x15	Present	Yes	-	Complete obliteration
20	Left Supraclinoid ICA	4.5x15	Present	No	-	Complete obliteration
21	Right Supraclinoid ICA	5.0x20	Present	No	-	Complete obliteration

Treatment timing and technical data

Eight patients were operated on on day 1 of the onset of SAH, seven were operated on on day 2, and six were operated on on day 3 (due to late arrival/referral to our center) (Table 2). DSA was performed on the same day of arrival at our center; two patients were operated on on the same day of arrival, and all the remaining patients were operated on within 48 hours after presentation to our center. All cases required a single FDD. Two patients required adjunctive balloon angioplasty (for achieving good vessel wall apposition), and none required coiling.

**Table 2:** Clinical course and follow-up data H&H: Hunt and Hess score; mWFNS: modified World Federation of Neurological Society score; GCS: Glasgow Coma Scale; PID: post ictus day; mRS: modified Rankin score

Case Number	H&H	mWFNS	GCS at Presentation	Presented on PID	Operated on PID	Discharged on PID	GCS at Discharge	Follow-up (Months)	mRS at Follow-up
1	2	2	14	2	3	10	15	30	1
2	1	1	15	1	2	8	15	18	0
3	2	3	13	2	3	9	15	24	2
4	3	1	15	3	3	9	15	20	0
5	2	1	15	2	3	10	15	24	0
6	1	1	15	1	2	9	15	20	0
7	2	3	13	0	1	8	15	18	1
8	2	2	14	0	1	7	15	18	0
9	2	1	15	2	3	9	15	16	0
10	1	2	14	1	2	9	15	15	0
11	2	1	15	0	1	8	15	16	0
12	1	1	15	1	2	7	15	14	0
13	2	1	15	0	1	6	15	14	0
14	2	2	14	0	1	6	15	12	1
15	2	1	15	2	3	7	15	12	0
16	2	1	15	0	1	6	15	13	0
17	1	1	15	0	1	6	15	14	0
18	2	2	14	2	2	7	15	14	0
19	2	1	15	2	2	8	15	13	0
20	1	2	14	1	2	7	15	12	1
21	1	2	14	0	1	6	15	12	0

Angiographic and clinical outcomes

**Figure 1:**
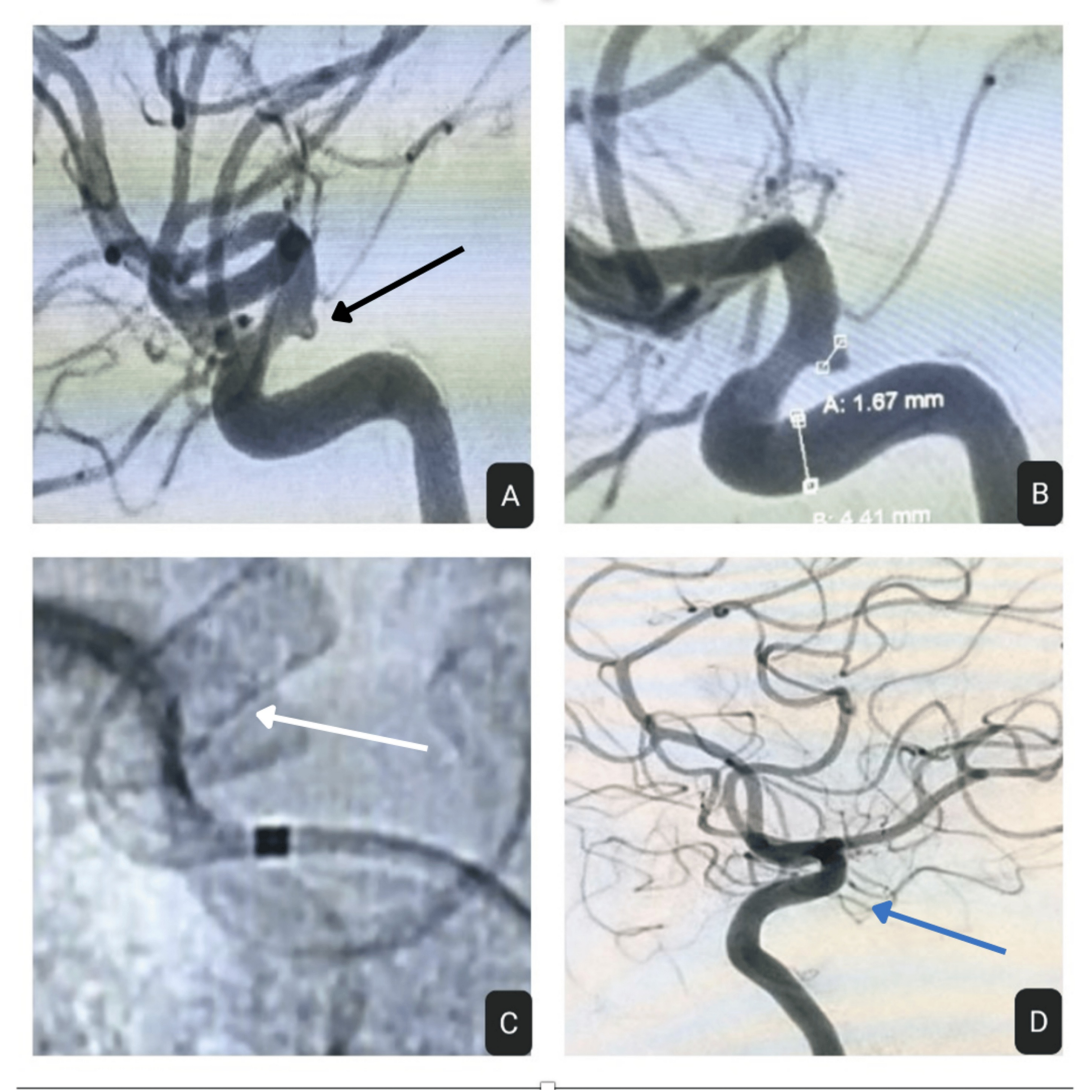
(A, B) Right supraclinoid ICA blister aneurysm (black arrow); (C) FDD placement (white arrow); (D) Complete obliteration seen at check angiogram done after six months (blue arrow) ICA: internal carotid artery; FDD: flow diverter device

**Figure 2:**
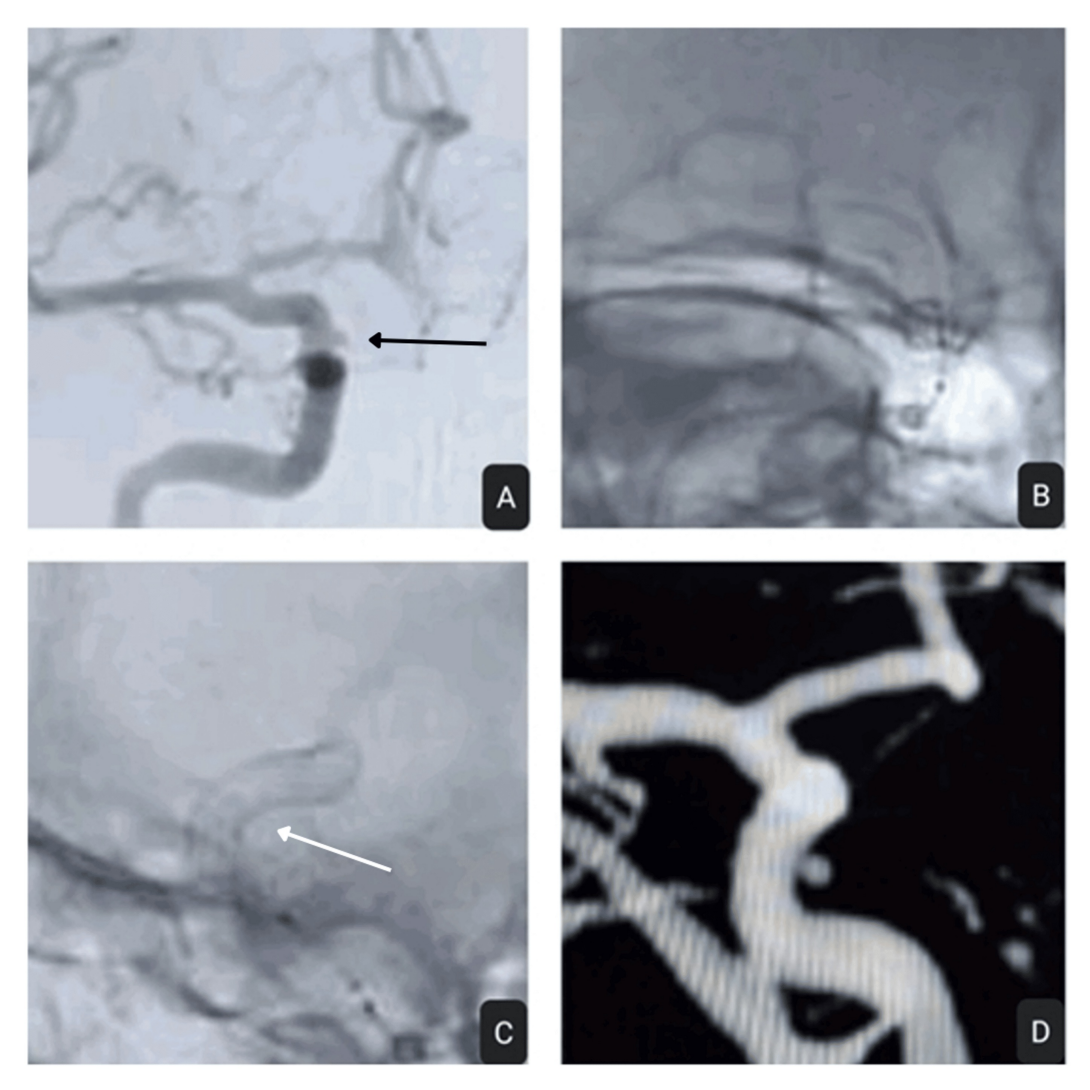
(A) Right supraclinoid ICA ruptured blister aneurysm (black arrow); (B, C) Post FDD deployment with good coverage of the neck (white arrow); (D) Partial obliteration seen in check angiogram done at six months ICA: internal carotid artery; FDD: flow diverter device

**Figure 3:**
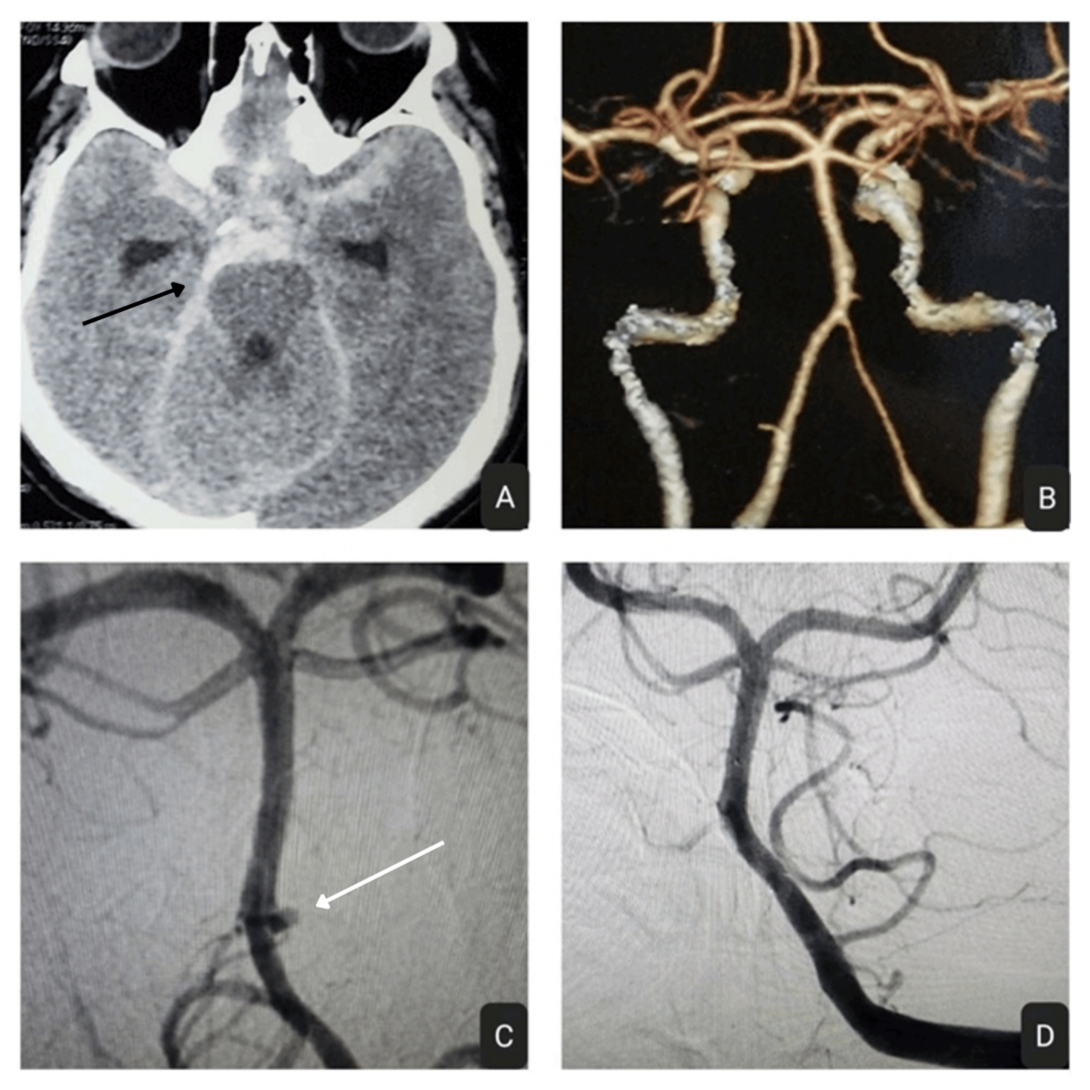
(A) CT Brain (plain) showing diffuse SAH (black arrow); (B) Proximal basila artery ruptured blister aneurysm seen on 3D CTA; (C) Diagnostic cerebral DSA; (D) Complete obliteration seen immediately after FDD placement SAH: sub-arachnoid haemorrhage; DSA: digital subtraction angiography; CT: computed tomography

Every patient underwent a post-procedure CT brain plain on the table to rule out contrast extravasation and a repeat non-contrast CT brain prior to discharge. The puncture site was checked after each procedure and rechecked after 48 hours to confirm no hematoma or puncture-related complications. No puncture site complication was noted in the study cases. Immediate change in contrast opacification, i.e., stasis, at the end of the procedure (15 minutes post deployment) was reported in 18 out of 21 (85.7%) cases. Immediate complete aneurysm obliteration was seen in nine (42.8%) patients. Two patients developed intraluminal partial thrombus in the FDD post deployment and received a 10 mg bolus of tirofiban followed by continuous intravenous infusion for 18 hours. None of the patients developed any post-procedure thromboembolic complications. Angiographic follow-up data were available for all patients and were obtained at six months after the treatment. Complete aneurysm obliteration was seen in 90.5% (19/21) of aneurysms (Figures 1-3). Two patients had an incomplete obliteration of the blister aneurysm at the six-month and one-year follow-ups. Clinically, all patients achieved favorable outcomes (mRS 0-2) at final follow-up. No perioperative mortality, rebleeding, thromboembolic complications, severe vasospasm, or hydrocephalus requiring CSF diversion was recorded.

## Discussion

Importance of early treatment

Blister aneurysms are most often diagnosed after an episode of subarachnoid hemorrhage. Although their size is small, they can substantially enlarge to a larger size and shape within days of presentation [6]. If insecure, they have a high incidence of re-rupture with a higher risk of fatal outcome. A comparison of the features of blisters and non-blister aneurysms of supraclinoid ICA indicates that two radiologic markers for accurate diagnosis are the distal angle of blood blister aneurysm (BBAs) and the dome/neck ratio <1 [14]. 

In order to partially compensate for the underlying medial deficit, the adventitia of intracranial arteries is thicker over bifurcation sites. Since blister aneurysms develop at non-branching artery segments, they lack this comparatively tight covering and are hence far more vulnerable [15]. They therefore have a higher risk of bleeding and a propensity for rapid development and rupture in the future [16]. Ogawa et al. reported the presence of dissection in 10 of their 40 blister aneurysms [6], while Satoh et al. observed a similar correlation in up to 16 of their 18 total patients [20]. Thus, all studies suggest early treatment of these ruptured blister aneurysms to avoid any further catastrophe, which formed the basis of our research.

Importance of 3D rotational angiography

It has been observed that single-slice CT angiography has a sensitivity of 25-64% when evaluating cerebral aneurysms less than 3 mm [21]. Conventional DSA, with its superior spatial resolution, continues to be the gold standard for the detection of cerebral aneurysms despite advancements in CT angiography. As such, it should be carried out whenever preliminary investigations yield negative results or to acquire a better understanding of aneurysm morphology. Three-dimensional conventional angiography was routinely performed in all our patients. In our series, five blister aneurysms (23.8%) were diagnosed on 3D conventional DSA after a 'negative' CT/MR angiography.

Effectiveness of endovascular flow diversion

Endovascular treatment (EVT) for blister aneurysms of the ICA is established as a safer and more suitable treatment approach than surgical approaches [22-24]. A thorough analysis of occlusion rates and clinical outcomes following FDD therapy of BBAs was conducted by Rouchaud et al. [24]. Of the 265 procedures they examined for ruptured BBAs, 62 involved the use of FDDs. In comparison to other endovascular techniques, they showed that FDDs had a greater occlusion rate of the BBAs without appreciably higher complication rates and comparable clinical outcomes [24]. Stent deployment, stent-assisted coiling, and flow diverter outcomes for BBAs were compared in a thorough analysis by Scerrati et al., published in 2021, that included 32 trials (684 patients, 707 BBAs) [25]. Overall, they recorded that EVT reconstructive procedures showed a 76.9% long-term total occlusion rate, an 8.9% intraoperative complication rate, a 76.6% satisfactory clinical result at final follow-up, and a 4.7% mortality rate.

A recent study concerning angiographic outcome showed that surgery was more likely to achieve early, complete occlusions, whereas there was an improvement of 32.4% between the early occlusion status and the follow-up occlusion status in the endovascular group [12]. This difference did not influence the outcome; thus, the clinical status at the outset has a much greater effect on the clinical outcome than the immediate complete occlusion of the aneurysm. We observed an increase in occlusion status from 42.8% (immediate) to 90.5% (follow-up) in our series.

The sole endovascular technique capable of reconstructing the vessel wall and sealing off any underlying defect is flow diversion, also known as endovascular parent artery reconstruction [26]. Immediately following deployment, the shear force on the aneurysm wall is removed, and blood inflow jets into the aneurysm are diminished. Progressive aneurysm thrombosis is brought on by this, and it often happens a few weeks after therapy. Flow diversion increases endothelial proliferation along the implanted stents while also preserving branching vessels, which is crucial for blister aneurysms because these lesions are typically found near the anterior choroidal or posterior communicating arteries [15].

Others have recently used both Silk stents and PEDs to treat ruptured blister aneurysms, with good clinical results (mRS 0-2 or GCS 4-5) in 66-94% of patients [27,28]. However, postoperative thromboembolic or hemorrhagic complications have been observed in 5-33% of cases, and procedure-related morbidity and mortality rates have remained as high as 15-25%. Following therapy, aneurysms treated with flow-diverting stents typically gradually occlude; full occlusion has been observed in 75-100% of cases at three to six months and in 94-100% of cases at 12 months [16,27-29].

Safety of DAPT protocols in the setting of acute aneurysmal rupture

The most feared complications of endovascular management are thromboembolic events (33.3%), followed by vasospasm (19%) [12]. In the setting of acute SAH, the risk-benefit profile of DAPT is considered carefully in deciding when to initiate such therapy. Kumar et al. have demonstrated that FDD with DAPT can be the treatment of choice in patients with blister aneurysms presenting with SAH [30]. The flow diversion procedures in acute SAH conditions were reviewed by Cagnazzo et al. [31]. They noted an overall complication rate of 18% and an acute occlusion rate of 35% in the BBA subgroup. Our series has shown an immediate stasis in 85.7% of patients with ruptured BBAs treated with FDDs, an immediate complete occlusion rate of 42.8%, and a complete occlusion of 90.5% at one year.

In our cases, we preferred ticagrelor over clopidogrel in acute settings only on the pharmacological basis. Asian populations are very prone to genetic polymorphism (*CYP2C19*), which is an important gene for hepatic metabolism of clopidogrel activation, which can lead to delayed action and decreased efficiency of the drug. On the other side, ticagrelor is fast-acting and does not need metabolic activation with reversible platelet inhibition, which is required for deploying high-metal coverage devices in acute settings. Thus, in our experience, use of DAPT, even in the presence of SAH, is safe and, in fact, mandatory to prevent thromboembolic complications. We did not encounter any intra- or postoperative re-rupture; however, two cases (9.5%) developed intraluminal thrombus post-FDD deployment, requiring additional antiplatelet bolus injection (tirofiban). These patients did not develop any embolic sequelae, so when immediate intervention is required, vigorous antithrombotic therapy is necessary for FDD stability, and the hemorrhagic risk of DAPT is acceptable.

Early flow diverter embolization is the key

According to recent studies, the overall complication rate is unaffected by the timing of flow diversion [31-33]. Tanburoglu et al. have shared their experience and demonstrated safety after early (<48 hours) treatment of ruptured blister aneurysms of the ICA with FDDs using SAPT [32]. In our study, all patients received treatment within 72 hours of the aneurysm rupture, and the outcomes were consistent with pertinent data found in the literature. The results we report support new research that suggests FDDs are the best vessel-preserving technique for subarachnoid hemorrhage, indicating that early therapy is safe and efficacious in the acute setting.

Limiations

Retrospective design, a single operator, and the absence of a control group mark the limitations of our study.

## Conclusions

Endovascular flow diversion represents a safe, effective, vessel-preserving option for the treatment of ruptured intracranial blister aneurysms. By interfering with the pulsatile flow into the aneurysm, the flow-diverting stent causes stasis, thrombosis, and shrinkage of the aneurysm, repairing it and reconstructing the parent artery. Our series supports early flow diversion as a viable vessel-preserving option in appropriately selected patients, but does not establish superiority over surgery or other EVT, given the absence of a control group.

With meticulous perioperative management and appropriate patient selection, early flow diversion with a DAPT protocol can minimize morbidity and mortality compared to surgical or alternative endovascular options. A larger series of patients will help to elucidate the difference in outcomes due to inter-user variability in the use of these devices for patients with ruptured blister aneurysms. 
